# Nursing students’ experiences of educational discrimination: a qualitative study

**DOI:** 10.1186/s12912-022-00925-y

**Published:** 2022-06-16

**Authors:** Zahra Hadian Jazi, Kazzem Gheybi, Zahra Zare, Hooman Shahsavari

**Affiliations:** 1 Nursing and Midwifery Faculty, Isfahan University, Isfahan, Iran; 2 Charles Perkins Center, School of Medical Sciences, University of Sydney, Sydney, NSW Australia; 3 Department of Operating Room, School of Allied Medical Sciences, Tehran University of Medical Sciences, Tehran, Iran; 4 Department of Medical-Surgical Nursing, School of Nursing and Midwifery, Tehran University of Medical Sciences, Tehran, Iran

**Keywords:** Justice, Discrimination, Nursing students, Educational system, Qualitative research

## Abstract

**Background:**

Although the need for justice and the elimination of injustice (or discrimination) is now a universally accepted principle, discrimination is still an unpleasant experience for many nursing students. This study aimed to explain the experiences of nursing students of educational discrimination and find out the main factors that cause this feeling.

**Methods:**

This is a qualitative study conducted in the nursing faculty of Shahr-e-Kord and the Iran university of medical science (IUMS) in Iran. Twelve nursing students were selected by purposeful sampling method and data were collected through face-to-face and in-depth interviews with semi-structured questions. All interviews were analyzed according to the content analysis method.

**Results:**

Three main themes and ten subcategories appeared. Extracted themes include: "inappropriate behavior of nursing professors (or instructors) " with 3 subcategories (1- discriminatory behavior by nursing professors (or instructors), 2- lack of sufficient self-confidence in nursing professors and transferring it to the student, and 3- the educator role in motivating or eliminating motivation); "Strict rules" with 3 subcategories (1- inequality in implementation of rights and rules among students of different disciplines, 2- differences in compliance with laws and regulations, and 3- nurses are being strictly monitored), and " Lack of nursing professional independence " with 4 subcategories (1- lack of authority, 2- lack of supportive organizations for nurses, 3- lack of proper social status of nursing in society, and 4- the high authority and power of physicians over other disciplines).

**Conclusions:**

In our study, it was shown that nursing students feel the most discrimination in front of medical students. Feelings of discrimination reduce self-confidence in nursing students. Therefore, nursing educators and professors must think of a solution, or at least they should not cause this feeling in them through inappropriate behavior and discriminatory speech and words.

## Background

Achieving professional values of nursing is a prerequisite for resolving conflicts, improving the quality of nursing care services, and increasing nurses' job satisfaction [1]. The core values accepted and endorsed by the American College of Nursing Associations (AACN) (1998) include human dignity, honesty, autonomy, altruism, and social justice, with social justice gaining so much attention in recent years. AACN defines social justice as fair treatment regardless of economic status, race, ethnicity, age, nationality, disability, or sexual orientation [2]. In fact, social justice, equitable distribution, facilities and resources for all people in a society is an abstract ideal that widely uses the concept of justice. But social injustice, is an incompatibility between what is and what should be. Edmond Cahn (1949) describes this concept as an emotionally charged structure that evokes anger, panic, shock and resentment .It prepares the humans or animals to resist attack. Social injustice, aroused by emotions arising from ethical cognitions about right and wrong, can motivate individuals, groups, and nations to take action, including violence and war, to correct perceived mistakes. [3].

Unfortunately, the issue of injustice or discrimination between the nursing and medical professions has always been accompanied by serious doubts and concerns [4]. The response of nurses to injustice is not always appreciated [5]. Indeed, physicians and nurses are considered the cornerstone of the healthcare system and their capabilities have a profound and important impact on the quality and efficiency of the medical system. Fair and non-discriminatory behaviors between physicians and nurses can increase job motivation, quality and satisfaction, and ultimately improve patient care [6]. Although in recent decades several quantitative and qualitative studies have been conducted to establish the concept of social justice among nursing graduates [7-10], it seems that academic nursing education has not been successful in this issue as Hosseinzadegan et al., (2021) in his studies indicates that lots of nursing students (in Iran) feel discrimination in both theoretical and practical courses [11].

Proper education plays a major role in educating justice-seeking nurses. Social justice and its importance in health care are among the nurses' curricula. Further attention to this issue in the practical and objective fields of education by educators can affect the thoughts, attitudes, and behaviors of students to pursue justice in health systems. However, unfortunately, the Iranian education system has failed to promote justice due to insufficient educational content, limited qualifications of nursing educators, and inappropriate educational approaches [11]. Given the importance of this issue, a qualitative approach was done to adopt a deep understanding of nursing students' experiences of educational discrimination. Therefore, the purpose of this study was to explain the perception and experiences of nursing students of educational discrimination and finding out the main factors that cause this feeling in nursing students in Iran.

## Methods

Because the concept of discrimination in nursing students is an unknown subject with different dimensions and causes, a descriptive and qualitative method was chosen to gain knowledge on participants' perceptions of the experience of discrimination. Therefore, qualitative methodology and content analysis method were used to better answer the question. Qualitative content analysis is a suitable method for analyzing written, verbal or visual messages that, among other qualitative methods, is not dependent on a specific philosophical point of view and therefore helps the researcher with sufficient flexibility in advancing their study [12].

Participants were selected by purposive sampling [13, 14] and those who could best enhance researchers' understanding of the phenomenon were employed. Inclusion criteria were: 1- Being nursing students (because the purpose of the study was to examine the experience of discrimination among nursing students); 2- Being the second to fourth year of bachelor, because in the first year they may have less experience of discrimination. It should be noted that Shahr-e-kord School of nursing had only a bachelor's degree at the time of the study; And 3- After explaining the purpose of the study, have informed consent to participate in the study. Researchers contacted students interested in participating in the study and made arrangements the time and place of the interviews with them, and if a student was reluctant to conduct the interview despite the previous announcement or did not wish to his information and experiences were mentioned in the study was excluded at any time of study.

Due to the qualitative nature of this study, the sample size was not determined before data collection. Therefore, the number of participants was decided based on the information collected. The end point or data saturation came when the new interviews did not provide additional information to the concept of purpose in the study. The analysis was performed simultaneously with data collection.

Participants included 12 undergraduate nursing students studying at Shahr-e-kord and Iran universities (in order to observe maximum diversity in the selection of participants).

Participants were first contacted and after explaining the purpose of the study, the date of the interview was determined. Data were collected through face-to-face and in-depth interviews with semi-structured questions at participants' favorite locations. Notes and field observations complemented the interviews. At the beginning of the interview, the interviewer asked a few introductory questions, followed by more specialized questions about the experience of discrimination. The interviews lasted about 40 min to an hour by one of the authors (ZH).

In this type of research, the text of the interviews is reviewed several times to be broken down into the smallest constituent and meaningful units (theme). After making a list of themes, and reviewing these words to clarify the similarities in their concept in order to place them in a subcategory based on the axis found between the themes, the same subtraction and inductive flow of the subclasses continued. These reviews, the sliding of the layers on top of each other, and the merging between the initial writings and the final classifications are repeated several times that the researchers eventually reach an acceptable stability between the data and a sense of satisfaction about the classes and subclasses [13]. Before the interviews, the participants were informed of the purpose and method of study, and researchers gained written informed consent from each participant and also researchers assured the participants that their personal information would be kept private.

In this study, Lincoln and Guba (1994) trustworthiness criteria was used [14, 15]. Therefore, in order to ensure the validity and scientific accuracy of the data, we used more than one method to collect data (such as field notes and interviews). Maximum diversity sampling (selection of participants from 3rd to 8th semester undergraduate students from Iran and Shahr-e-kord universities) was also applied. Long-term engagement with participants and reviewing interviews before analyzing them, reviewing and modifying the codes several times, reviewing the codes with all members of the research team, and with the participants. The recorded interviews were analyzed by the researchers (ZH and HSH) according to the qualitative content analysis method and using the MAXQDA version 10.

## Results

Twelve undergraduate nursing students participated in this study. The mean age of participants was 21 years (19 to 24 years). Sixty-six percent of the participants were girls and 34% were boys. Seventeen percent of the students were second-year (semesters 3 or 4), 58% were third-year (semesters 5 or 6) and 25% were fourth-year (semesters 7 or 8). Because the main interviewer of this study was in the Shahr-e-kord university, 65% of the participants were selected from Shahr-e-kord university and 35% from the university of Iran.

Participants in this study shared their experiences of educational discrimination. During data analysis, three themes and 10 subcategories appeared. The extracted themes and classes focus on the reasons that are caused nursing students to feel discrimination, which is described below (Table 1).

**Table 1:** The extracted themes and classes focus on the reasons that are caused nursing students to feel discrimination

Inappropriate role of nursing professors (or instructors)	Discriminatory behavior by nursing professors (or educators)
	Lack of sufficient self-confidence in nursing professors and its transfer to the student
	The educator role in motivating or eliminating motivation
Strict rules	Inequality in supervising and implementation of rules among students of different disciplines
	Differences in compliance with laws and regulations
	Nurses are being strictly monitored
Lack of nursing professional independence	Lack of nursing authority
	Lack of supportive organizations for nurses
	Lack of proper social status of nursing in society
	The high authority and power of physicians over other disciplines

### Theme 1: inappropriate role of nursing professors (or instructors)

#### Discriminatory behavior by nursing professors (or instructors)

In this regard, many students considered the reason for the feeling of discrimination is the behavior of their professors with students who are all on the same level in terms of seniority (both in the field of teaching theory and in the field of clinical education in the hospital). Regarding this theme expressed one of the participants:

“At the beginning of the classes, after entering to the nursing faculty, the professor said: You had a very bad last year and you were not successful at all, and it made you come to a field (nursing) that is not a good job for your future at all …". Because one of his own children is studying medicine. (Participant two).

Some students believed that the behavior of professors in the theoretical and clinical classes was different and sometimes even contradictory. For example, a student said, "it is written in nursing textbooks that a nurse should first, have a strong relationship with the patient so that he or she is comfortable. So, the lessons and rules give us that authority. but I personally went up to one of the patients and greeted him warmly. Then the professor came and said why did you become intimate with the patient? … Go very formally, ask questions and come back …." (Participant two).

#### Lack of sufficient self-confidence in nursing professors and its transfer to the student

Many students believed that their instructors do not have enough self-confidence and authority in the internship environments and show weakness in the face of medical students and their professors, which cause others to dare to discriminate between nursing students and other disciplines. In this regard, a student said:

" If a fellowship comes in the patient’s room with their students, our instructors tell us -in front of the medical students- to go out and we will come back later. They tell us not to argue, the room is for them (medical students) (Participant eight).

Another student said:” It is upsetting that if while we’re using a classroom and medical students also want to use the room, they have the authority to make us leave the class, and our instructor is not so confident to not let this happen! “(Participant seven).

#### The educator role in motivating or eliminating motivation

Students stated that instructors have an important role in motivating students and make them to work and study more enthusiastically, but unfortunately in many cases they are being compared with medical students and this causes them to lose motivation and self-confidence to continue their education and feel ashamed of being a nurse.

In this regard, a student said: "We were in the second semester … Our professor told us, what field would you like to learn more about? Ask me some questions about it. Some students said that we would like to know more about pharmacology, so that if a doctor ordered a medication, and we thought that the medication interfered with another or it was not appropriate, we could mention it to them or we would like to be so good to know what is good for the patient now and to do it ... The instructor said: you never can do that... (Participant seven).

In addition, another student said, "One of the professors said: each of you who does not like nursing has to go and study medicine. I think medicine is much better!' (Participant five**)**

### Theme 2: Strict rules

#### Inequality in supervising and implementation of rules among students of different disciplines

Violation of the rules by medical students and failure to deal with them, lack of supervision of medical students, excessive supervision of nursing students, failure to comply with the rules in the field of medicine, etc. were cited by many students as a source of discrimination. For example, a student described his experience of discrimination in this regard: "There is no rule for medical students. There may be rules, but there is no supervision on them. They go to the hospital anyway they want. Sometimes they do not even wear a uniform"(Participant five).

#### Differences in compliance with laws and regulations

The participating students believed that the level of compliance with university rules and regulations and the clinical environment of hospitals were not the same simply because the disciplines were different. A student considered the extent of compliance with hospital rules and regulations not only in their dressing code, but also in the hospital's clinical regulations and communication with the patient, saying: "For example, a medical student can talk to patients in any way they want and they have the right to treat the patient as they wish, but if nursing student greet warmly with patients, they will face punishments from their staff and trainers" (Participant two).

#### Nurses are being strictly monitored

The students stated that so many rules and regulations for nurses and nursing students have been defined in detail and that non-observance of them has bad consequences that it seems that this group is under a microscope! One student described his experience as "from the very beginning there are certain rules for nursing that do not exist for any other field. For example, the length of the uniform should be that inches below the knee or be so loose; pants should not be jeans at all; the fabric of the pants should be either black or blue, etc.… Everybody can understand from the student's appearance what field they are, nursing or medical?! "(Participant two).

Another student said, "Doctors are not strict at all, and that in itself is discrimination" (Participant six).

### Theme 3: lack of nursing professional independence

#### Lack of authority

Doctors' interference in nurses' affairs and nurses' non-objection, being dependent on the doctor, reducing the quality of nursing care due to the nurse's unquestioning obedience to the doctors, etc., were all points that student mentioned and will eventually lead to feelings of discrimination.

One student said: "if nurses object to the doctors, in many cases, it has very bad consequences for them, so look, we do whatever they say... and whatever the doctor orders, we do... and what does this mean? It means that the genius of the nurse goes blind "(Participant seven).

#### Lack of supportive organizations for nurses

The students said that another factor that causes nursing to lack a proper professional independence and as a result suffer a lot of discrimination from others, are their lack of support for each other.

In this regard, it can be attributed to factors such as the feeling of futility of nursing liability insurance, lack of defense support for the nurse, consider a low worth of the nurse's work even in the nursing system, different behavior of even nursing staff with nursing students in front of medical students, not appreciating the work of nursing students, etc.

A student complained about the behavior of nursing staff towards nursing students, saying, "Even nursing staff do not respect us, they don’t consider us as their future coworkers" (Participant four).

#### Lack of proper social status of nursing in society

Students consider that one of the things that causes discrimination is the lack of a proper social position for nursing in society and the view of all people. Regarding the role of the media in creating a negative attitude towards the nursing profession, one student said: "In TV movies, nurses are always changing sheets, or saying yes to doctors’ orders...! Or even in the novels I read the doctor is always in a superior position, but the nurse always is insulted! these things change the people’s attitudes towards nursing” (Participant nine).

The same student commented about the negative attitude of people in the community, including patients, towards nurses: "It means that when you tell patients that I am a nursing student, they will not answer you anymore ... but if a doctor comes, everyone will respect them" (Participation nine).

#### The high authority and power of physicians over other disciplines

Another factor that has reduced the authority and professional independence of nurses is the high authority of medical practitioners in the Iranian society, which has led to a sense of superiority over other disciplines. A student said: “I heard it myself that a nursing supervisor objected to an intern medical student. But what happened eventually?... The nursing supervisor went to the head of the hospital and apologized" (Participant seven).

Or another student said: "A nurse in the hospital cannot show genius and creativity in any way; even if it is true, the head nurse or the supervisor says: how dare you to interfere in the treatment of the patient?" (Participant fifteen). " But in other countries, multidisciplinary teams are gathered including a nurse and decide for the treatment of a patient”,", he added "Such things will not happen in our society (Iran) for another thousand years! Because there is only a medical commission and they do not consider the nurse, even if she has a lot of knowledge" (participant seven)

## Discussion

The aim of this study was to explain the perception and experiences of nursing students of educational discrimination and finding out the main factors that cause this feeling in nursing students in Iran.

Three main themes and ten subcategories appeared. Extracted themes include: inappropriate behavior of nursing professors (or instructors), Strict rules and lack of nursing professional independence which some of more important findings are discussed in details.

,In this study, it was found that one of the factors that instigates a sense of discrimination in nursing students was the inappropriate behavior of educators and clinical instructors in the university educational environment and then in the hospital. The students stated that from the very first semesters, their professors cause nursing students to lose their motivation by comparing the field of nursing with medicine and makes them become disenchanted with their profession. In addition, many students feel insignificant in compared with medical students after entering the clinical environment. because nursing instructors discriminated against them or they were supported in front of the hospital staff. In fact, the attitudes and behavior of the instructor can play a great role in motivating a student to work in clinic setting or turn them away from their field, which in itself is closely related to the instructor's vision of nursing. Certainly, a nursing educator who believes in his profession and is passionate about nursing will also indoctrinate this interest to his students. In fact, student support by the instructor increases students' confidence and motivation, learning, professional development, and positive outcomes [16]. Therefore, according to the findings of this study, it is suggested that nursing school authorities be more careful in selecting nursing instructors and professors, and hire people who not only are impeccable academics, but are also passionate with the field. Consistent with our results, other studies showed that effective educators are important as a key strategy as well as a facilitator of education in empowering and supporting nursing students" [17,18]. In a study by Witzel et al. (2008), it was found that the support of the faculty and instructors were the most important source of support for nursing students during their studies [19]. Therefore, officials should not ignore the importance of selecting efficient, effective, and experienced nursing and instructors.

In this study, another factor that created a sense of discrimination among students was the existence of very detailed and strict rules for nursing students that made them feel that their behavior and even their physical presence were under constant control. This feeling was intensified when they saw the lack of attention and supervision on other students (especially medicine). We believe that the reason for this is the atmosphere in Iran hospitals where there is a superiority of medical practitioners. In fact, medical practitioner has a dominance in Iran healthcare system in terms of income and authority compared to other professions [6]. This monopoly, may cause many staff, especially nurses, to feel incompetent and it might affect the quality of their performance. In this regard, the study of Aiken et al. showed how the idea of being under the control of physicians has affected job satisfaction and professional identity of nurses and reduced the quality of patient care [20]. Roxburgh and his Colleagues also reported that nursing students need to feel empowered in order to efficiently study, and function in a clinical environment. This feeling is formed in an environment which the student feels belonged and supported by other nurse’s health care staff and not that only nurses have to enforce the rules and there are no rules for the rest [21]. Unfortunately, few studies have been done to compare compliance with laws and regulations between different disciplines, so this study may be a good start to address this issue.

Another issue mentioned was the lack of professional independence in the field of nursing which may instigate much of the discrimination feeling. The results of our study suggest an unconscious lack of self-confidence in nursing professors towards medical professors and students. The existence of facilities and educational classrooms available in the hospitals exclusive to medical students, involvement of physicians in matters related to nursing and even ordering and forbidding them in some issues, the lack of a proper professional position in society and among patients and other issues, all of which indicate the lack of independence and professional identity of nursing. Quoting du Toit, stated that having independence in the clinical activities of leadership and management power in the health care system, skills, critical thinking and commitment to the profession is important for the formation of a professional identity in nursing [22].

Impaired professional independence can affect the learning process of nursing students. But having a positive professional identity and independence can increase nurses' confidence in quality nursing care. However, this picture contrasts with what nurses see in practice. Nursing students often encounter professional identity problems in their own learning process. Students who do not experience a positive professional identity will experience lower self-esteem, weakened clinical reasoning skills, experienced many problems in interpersonal communication, and a sense of belonging to the nursing profession [23]. This will lead to less flexibility and efficiency when facing healthcare challenges, and being aware of these challenges and problems will be important for students' ability to learn and perform care tasks [23, 24]. Hovere et al., Stated that the nature of nursing has not always been clear and that nurses continue to suffer from stereotyped statements about nursing. These public stereotypes can create a stressful environment in health care systems [25]. Therefore, nurses and nursing students should do a high-quality care work to improve the negative image of the profession, increase public awareness about their various roles and advanced nursing positions, and to be better seen, they can use their power through the media [26].

### Implications for research/practice

The issue of justice and in contrast to injustice is a very important issue in education. This study tried to depict a corner of the feelings of nursing students who had experienced discrimination in the educational environment and its effect on self-confidence, job satisfaction, motivation and ultimately the quality of patient care. The findings of this study can be used extensively by nursing professors and educators to at least not cause this feeling in their students and avoid instigating the feelings of humiliation and frustration of nursing students in the future. In addition, professors of other disciplines and anyone involved in education can put the findings of this study at the forefront of their work and remember that all their actions, behaviors and words during theoretical and clinical teaching are carefully analyzed by students and can increase motivation and job satisfaction in the future or, conversely, cause frustration and despair.

### Limitations of study

One of limitations of this study- like all qualitative studies- was the consideration of the two nursing faculties (Shahr-e-Kord nursing faculty and Iran Nursing faculty in Tehran) and two teaching hospitals in Iran. As such, it may not be a representative of the experiences of all the nursing profession members in Iran. In addition, it is highlighted that this study is based on qualitative data and thus the aim was in-depth understanding rather than reaching statistical generalization. Limitations of our study proposed the need for conducting further studies with larger and mixed groups and in different cultures.

## Conclusion

In our study, it was shown that nursing students feel discrimination in many cases, and because most of the groups that cooperates and interacts with nurses are doctors and medical students, the feeling of discrimination particularly stems from medical practitioners and students. Feelings of discrimination in many cases cause a lack of interest and motivation to choose nursing or reduce self-confidence in students. This can negatively affect training of efficient and professional nurses for the future and cause problems for the health and treatment system. Therefore, it is necessary for nursing educators and professors to avoid causing the feeling of discrimination by inappropriate attitudes towards nursing students. More studies are required to elucidate the reasons of injustice in clinical education environment and finding a solution to this issue.

## Data Availability

We do confirm that all data generated or analyzed during this study are included in this published article.
